# General planar transverse domain walls realized by optimized transverse magnetic field pulses in magnetic biaxial nanowires

**DOI:** 10.1038/srep43065

**Published:** 2017-02-21

**Authors:** Mei Li, Jianbo Wang, Jie Lu

**Affiliations:** 1 School of Physics and Technology, Center for Electron Microscopy and MOE Key Laboratory of Artificial Micro- and Nano-structures, Wuhan University, Wuhan 430072, China; 2 College of Physics and Information Engineering, Hebei Advanced Thin Films Laboratory, Hebei Normal University, Shijiazhuang 050024, China

## Abstract

The statics and field-driven dynamics of transverse domain walls (TDWs) in magnetic nanowires (NWs) have attracted continuous interests because of their theoretical significance and application potential in future magnetic logic and memory devices. Recent results demonstrate that uniform transverse magnetic fields (TMFs) can greatly enhance the wall velocity, meantime leave a twisting in the TDW azimuthal distribution. For application in high-density NW devices, it is preferable to erase the twisting so as to minimize magnetization frustrations. Here we report the realization of a completely planar TDW with arbitrary tilting attitude in a magnetic biaxial NW under a TMF pulse with fixed strength and well-designed orientation profile. We smooth any twisting in the TDW azimuthal plane thus completely decouple the polar and azimuthal degrees of freedom. The analytical differential equation describing the polar angle distribution is derived and the resulting solution is not the Walker-ansatz form. With this TMF pulse comoving, the field-driven dynamics of the planar TDW is investigated with the help of the asymptotic expansion method. It turns out the comoving TMF pulse increases the wall velocity under the same axial driving field. These results will help to design a series of modern magnetic devices based on planar TDWs.

A magnetic domain wall (DW) is the intermediate region separating adjacent magnetic domains with different orientations. The finite DW width comes from the competition between the magnetocrystalline anisotropy and the exchange interaction. For two-dimensional (2D) magnetic films, the even parity of the magnetocrystalline anisotropy generally results in an angular rotation of 180° of the magnetization vector in a DW. Among these 180° walls, two types are of great interests: Bloch walls and Néel walls. They are the simplest cases and can be analyzed theoretically. In a Bloch wall, the magnetization vector rotates in the DW plane (interfacial plane between adjacent domains) and only generates surface magnetic charges. While for a Néel wall, the rotation plane of the magnetization is perpendicular to the DW plane, thus resulting in volume charges. Therefore Bloch (Néel) walls appear in thick (thin) magnetic films due to the energy minimization strategy. In reality, the magnetostatic interaction greatly complicates the situation and results in vortex or other more sophisticated wall structures. Traditionally, the magnetization dynamics is described by the nonlinear Landau-Lifshitz-Gilbert (LLG) equation^1^, in which the damping coefficient is phenomenologically introduced. Mathematically, DWs are soliton solutions of the LLG equation. They are characterized by a topological charge index^2^ and protected by a finite energy barrier against the trivial single-domain state, thus can not be continuously deformed to it.

Another interesting topological non-trivial entity in 2D magnetic films is the skyrmion, which is a stable topological object with particle-like properties in numerous field theories. It was first proposed as a model of baryons in nuclear physics^3^ and was first experimentally observed in quantum Hall ferromagnets^4^. Skyrmions were also observed in helimagnets^5^, in which the inversion symmetry is broken, and stabilized by the Dzyaloshinskii-Moriya interaction (DMI)^6^,^7^. Most recently, they were proposed as the basic units of the next-generation magnetic memory devices^8^. The topological feature of an isolated skyrmion is characterized by the quantized winding number (skyrmion number), which describes how the electron changes its spin when passing through the skyrmion core^9^. Two typical kinds of skyrmions are of special interests: the azimuthal^10^ and the radial^11^ skyrmions. Although skyrmions and DWs are different topological non-trivial entities, the spin textures in them can both be mapped onto a unit sphere, thus leading to a mapping connection between each other. More intuitively, when an azimuthal (radial) skyrmion is cut from the core along an arbitrary radial direction, one will obtain a structure similar to a Bloch (Néel) wall. This explains why an azimuthal (radial) skyrmion is also called a Bloch (Néel) skyrmion.

In the past decades, substantial improvements in film preparation and etching technology have generated a new class of physical systems: magnetic nanowires (NWs). Based on them, magnetic nanodevices, such as the domain-wall (DW) logics^12^, racetrack memories^13^, and shift registers^14^, etc., have developed rapidly. Advances in manufacturing thinner NWs greatly improve the integration level of these devices and make them quasi one-dimensional (1D) systems. Therefore, transverse DWs (TDWs) dominate^15^,^16^. For 1D NWs, generally the easy axis (or effective easy axis from magnetostatic interaction) coincides with the wire axis, which leads to head-to-head (HH) or tail-to-tail (TT) Néel-like TDW configuration. However, as we will show below, the tilting plane (in which the magnetization rotates) of the static TDW will be confined within the easy plane and cannot be arbitrarily controlled.

To manipulate the TDW tilting attitude, using a uniform transverse magnetic field (TMF) is the easiest way and has been intensively studied^17^,^18^,^19^,^20^,^21^. However, a uniform TMF have two influences. First it pulls the magnetization out of the wire axis in the two domains and thus the TDW is not a 180° wall any more. Second, it induces a twisting in TDW azimuthal distribution^21^. For application in high-density NW devices, it is preferable to erase the twisting so as to minimize magnetization frustrations and stochastic fields. In this work, we smooth the TDW twisting by changing the TMF from uniform to space-dependent. We focus on the case where the TMF strength is fixed and its orientation is allowed to change freely. For statics, we will provide an optimized TMF profile that maintains a planar TDW with arbitrary tilting angle.

Besides the statics, the TDW dynamics is also attractive since it directly leads to fascinating inspirations of magnetic nanodevices in modern information industry. In magnetic NWs, TDWs can be driven to propagate along wire axis by magnetic fields^22^,^23^,^24^,^25^,^26^,^27^, spin-polarized currents^28^,^29^,^30^,^31^ or temperature gradient^32^,^33^,^34^, etc. Among them, the field-driven case is the most basic. The Walker’s analysis^22^ about field-driven DW dynamics based on the LLG equation indicates the crucial role of the transverse magnetic anisotropy of a NW, which leads to the “Walker limit” separating two distinct propagation modes: traveling-wave and reciprocating rotation. Meantime the TDW tilting plane departs from the easy plane and/or even rotates about the wire axis. Usually the traveling-wave mode attracts most attention since in this mode the DW propagates as a rigid body, bearing a velocity proportional to the driving field and inversely proportional to the damping coefficient. Alternatively, the reciprocating rotation mode is less interested due to the back and forth manner of the DW motion which greatly reduces its velocity. In this work, with the help of the asymptotic expansion method, the planar TDW dynamics carrying the corresponding well-designed TMF profile along with it is investigated. It turns out that the planar TDW will acquire higher velocity than that under pure axial driving field in the traveling-wave mode from Walker’s analysis.

## Results

**Figure 1:**
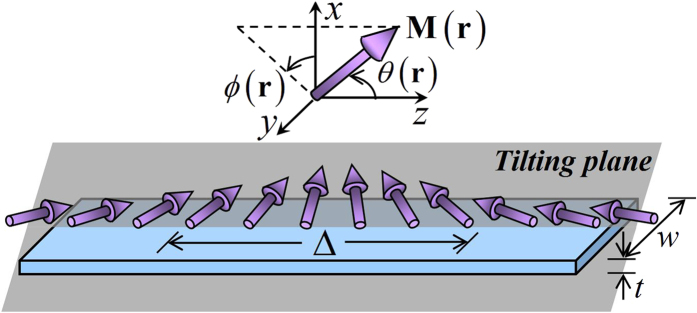
A HH TDW with width Δ in a nanowire with thickness *t* and width *w*. The coordinate system is set as: the *z*–axis is along wire axis, the *x*–axis is in the thickness direction and 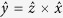. The magnetization vector 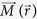 is fully described by its polar angle 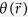 and azimuthal angle 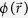. In particular, the wall is called a planar TDW, when the magnetization vector therein rotates in a plane called the “tilting plane”.

The system is sketched in Fig. 1. A HH TDW with width Δ is nucleated in a thin enough magnetic NW with thickness *t* and width *w*. The *z* axis is along wire axis, the *x* axis is in the thickness direction and 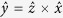. The magnetization 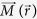 with constant magnitude *M*_*s*_ is fully described by its polar angle 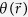 and azimuthal angle 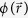. A TMF profile with fixed strength *H*_⊥_ and tunable orientation angle Φ_⊥_(*z*),


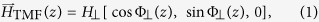


is applied across the whole NW.

The time evolution of 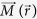 is described by the LLG equation,


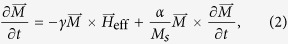


where *γ* is the gyromagnetic ratio, 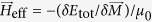 is the effective field and *α* is the phenomenological damping coefficient. For the biaxial NW under investigation, the total magnetic energy density is


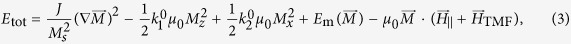


in which *J* is the exchange coefficient, 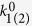 is the dimensionless crystalline anisotropy coefficient in the easy (hard) axis, 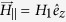 is the axial driving field and *E*_m_ is the magnetostatic energy density which is usually quite complicated due to the nonlocal nature of dipole-dipole interaction.

### Planar TDW in easy plane without any external fields

In thin enough NWs with regular shapes, as we have illustrated before^21^, most part of *E*_m_ can be described by quadratic terms of *M*_*x*,*y*,*z*_ in terms of three average demagnetization factors *D*_*x*,*y*,*z*_, thus 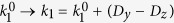 and 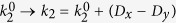. In this sense, the nonlocal magnetostatic energy is mimicked by local quadratic energy terms. This simplification neglects most magnetization frustrations, however will be quite good when the NW becomes a quasi 1D system. For 1D systems, 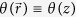, 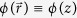 hence 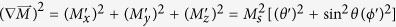 where a prime means spatial derivative to *z*. In the absence of any external fields, the total magnetic energy is


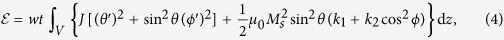


in which we have redefined the energy origin by dropping “
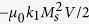
” with *V* being the wire volume.

To obtain a stable magnetization configuration, we need to minimize 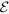. First one should let 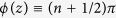 to eliminate (*ϕ*′)^2^ and cos^2^ *ϕ* terms. This makes the magnetization vector lie in the easy plane everywhere. Then the wire will have minimum energy when 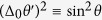 where 
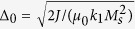
. Obviously, the single-domain solution 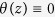 (or *π*) is trivial. Now we focus on DW solutions. For a HH DW, the boundary conditions are *θ*_*z*=−∞_ = 0, *θ*_*z*=+∞_ = *π*. While for a TT DW, they are *θ*_*z*=−∞_ = *π, θ*_*z*=+∞_ = 0. Combing with the above boundary conditions, the resulting *θ*–profile is the well-known Walker’s solution^22^,


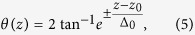


with *z*_0_ being the TDW center and + (−) means HH (TT) TDW. In brief, for a thin enough biaxial NW, the stable TDW is a planar wall which lies in the easy *yz*–plane.

### General static planar TDWs

In this work, we aim to realize a complete planar TDW with arbitrary tilting attitude. For simplicity, we focus on HH TDWs (TT TDWs can be investigated similarly). To achieve this, we need a TMF to pull the azimuthal angle plane out of the easy plane. However, a uniform TMF generally induces twisting around the TDW center^21^. To erase the twisting, we fix the TMF strength and allow it rotate freely to look for an optimized profile that results in a planar TDW.

First we rewrite the vectorial LLG equation (2) to two coupled scalar equations,


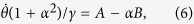


and


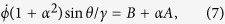


with


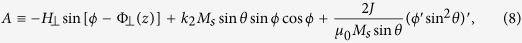



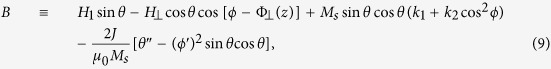


where a dot means time derivative.

To realize a static planar TDW, first we need the magnetization orientations in the two faraway domains. In the left domain (*z* → −∞), the polar (azimuthal) angle of magnetization is denoted as *θ*_∞_ (*ϕ*_∞_), while those in the right domain (*z* → +∞) are *π* − *θ*_∞_ and *ϕ*_∞_, respectively. The static condition 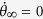, 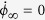 and domain condition 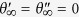, 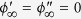 turn equations (6) and (7) to


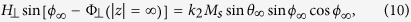


and


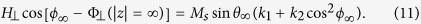


The solutions of the above two equations are


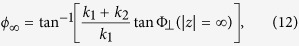


and


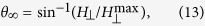


with


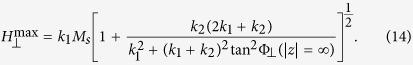


From equation (12), a necessary condition of the TDW being planar is 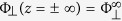. Without losing generality, suppose 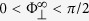, we have 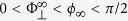. In addition, from equation (13) the TDW existence condition (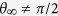) sets an upper limit of the TMF strength,


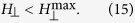


Next we move to the TDW region. The static condition *A* = *B* = 0 becomes


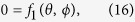


and


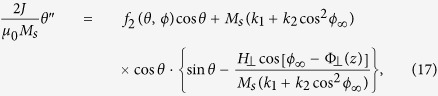


with


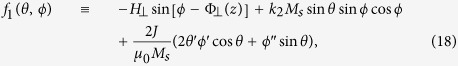



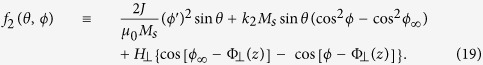


Now we consider a complete planar TDW


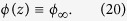


Obviously, this solution makes *f*_2_(*θ, ϕ*) = 0 and thus


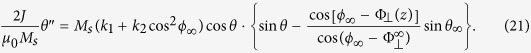


On the other hand, *f*_1_(*θ, ϕ*) = 0 is reduced to


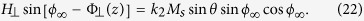


Comparing equation (22) with equation (10), we obtain the dependence of TMF orientation on TDW polar angle,


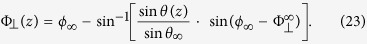


or vice versa. Equation (23) shows that the TMF cannot be uniform. It also requires 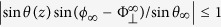, which sets a lower limit of the TMF strength,


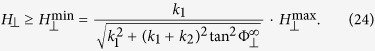


Put equation (23) back into equation (21), we then have


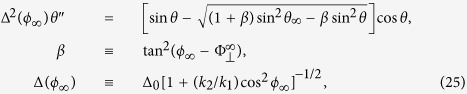


where *ϕ*_∞_ and *θ*_∞_ are given by equations (12) and (13) with 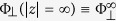.

When 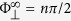, 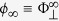 hence *β* = 0. Equation (25) is reduced to a Walker-ansatz-like form,


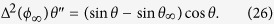


Its solution has been presented by equation (16) in our previous work^21^.

For the more general case where 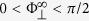, obviously *β* > 0 and equation (25) is not a typical Walker-ansatz form. Actually *β* measures the deviation between our profile and the classical Walker’s solution. Simple calculus yields when 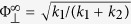 and 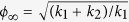, *β* is maximum and turns out to be “
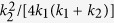
”. After some algebra, we have


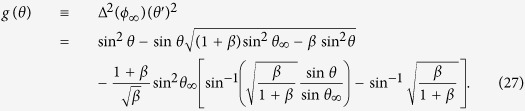


When *z* ≥ *z*_0_, *θ* ≥ *π*/2. From equation (27), in principle the following integral


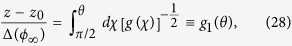


gives the right-half profile 
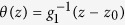
. For the left half with *z* < *z*_0_, one has *θ*(*z* − *z*_0_) = *π* − *θ*(*z*_0_ − *z*) due to the symmetry about the TDW center. Put it back into equation (23), the corresponding TMF orientation profile is obtained.

Summarizing the above analytics, we propose the algorithm of realizing a rigorous planar HH TDW with an arbitrary tilting angle *ϕ*_∞_ (TT TDWs can be obtained similarly):

1. Given *ϕ*_∞_, equation (12) gives the TMF orientation 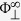 in the two domains and hence the parameter *β* in equation (25),
2. Equations (15) and (24) provide the upper limit 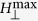 and lower limit 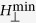 of the TMF strength,
3. For an allowed *H*_⊥_ (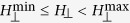), equation (13) gives the TDW boundary condition *θ*(*z* = −∞) = *θ*_∞_ in the left domain, while that in the right domain is *θ*(*z* = +∞) = *π* − *θ*_∞_.
4. Based on the above boundary conditions, equation (28) gives the final *θ*–profile of this planar TDW,
5. The corresponding TMF orientation distribution responsible for this planar TDW follows equation (23).

From the above algorithm, given TDW tilting attitude *ϕ*_∞_ in principle we are able to realize a variety of planar TDWs with different boundary conditions originating from different choices of TMF strength.

**Figure 2:**
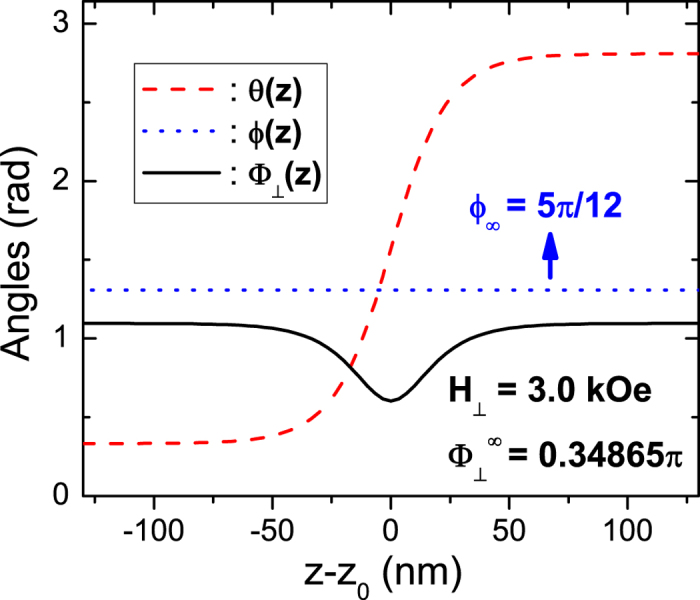
Example of a general planar TDW (neither in easy nor in hard planes) and the corresponding TMF orientation profile in a 5 nm × 100 nm × 10 *μ*m biaxial nanowire. The magnetic parameters of the wire are: *M*_*s*_ = 500 kA/m, *J* = 40 × 10^−12^ J/m, 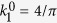, 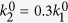, and *α* = 0.01. The TDW tiling angle is selected as *ϕ*_∞_ = 5*π*/12 (blue dotted line). This leads to 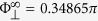, 
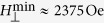
 and 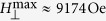. The polar profile of the TDW for *H*_⊥_ = 3 kOe is shown by the red dashed curve and the corresponding TMF orientation profile is indicated by the black solid curve.

Next, we illustrate our algorithm in a 5 nm × 100 nm × 10 *μ*m biaxial NW. For this wire geometry, the three average demagnetization factors are: *D*_*x*_ = 0.92793, *D*_*y*_ = 0.07140, *D*_*z*_ = 0.00067. The magnetic parameters are: *M*_*s*_ = 500 kA/m, 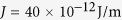, 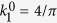, 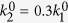, and *α* = 0.01. After the “nonlocal-to-local” simplification to the magnetostatic interaction, we have the following total anisotropy coefficients: 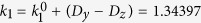 and 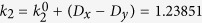. In this wire, we want to realize a planar TDW with tilting plane 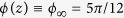 (blue dotted line in Fig. 2). We perform our calculation following the above algorithm. First, the TMF orientation in the two domains should be 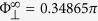 hence the parameter *β* is 0.047. Second, the upper and lower limits of the allowed TMF strength are 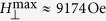 and 
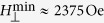
. Different choice of TMF strength will result in different polar angle profile of the planar TDW. In step 3, we take *H*_⊥_ = 3 kOe as an example. Then the polar angle in the left domain is *θ*_∞_ = 0.1060*π*, while that in the right domain is 0.8940*π*. Based on them, in step 4 the *θ*–profile of the planar TDW is calculated from equation (28) and indicated by red dashed curve in Fig. 2. Fifth and finally, the orientation profile of the TMF pulse responsible for this planar TDW is shown by black solid curve in the same figure. Obviously, to maintain the planar TDW, the TMF vector should be closer to the hard *xz*–plane around the TDW center to resist its twisting trend.

The above analytics completely describes a single planar TDW inside a biaxial NW. However, for real application in DW-based magnetic racetrack devices, a series of TDWs (HH and TT walls appear alternately) will be nucleated in a NW (generally biaxial since the magnetostatic interaction would induce an effective hard axis in transverse direction). If the TDW widths are quite small compared with the domain lengths, our analytics can be applied to each TDW independently and a series of planar TDWs with either identical or various tilting attitude can be realized by applying the corresponding TMF pulses segmentally. On the contrary, if the TDW widths are comparable with the domain lengths, the above analytics fails. When the TDWs are distributed evenly, similar to electrons in crystalline potential, a solution with Bloch-wave-like form should be appropriate, however beyond the scope of this article.

At last, although our analytics is performed in magnetic biaxial NWs, it can be easily generalized to other magnetic systems, such as magnetic films with periodic 180° stripe domains^35^,^36^, or exchange coupled magnetic hard/soft/hard sandwiched systems^37^. For a magnetic film with periodic 180° stripe domains, it can be viewed as a quasi-1D magnetic system. When the film is thin enough, HH or TT Néel walls separate the adjacent domains. Then the above discussion about a series of planar TDWs in racetrack NWs also applies in this situation. For a sandwiched hard/soft/hard magnetic system coupled by exchange interaction, when analytically solving the LLG equation for planar TDW solutions, besides the boundary conditions in the two faraway domains in hard materials, the magnetization distribution must satisfy the interfacial constraints at the two interfaces originating from the variation of the surface contributions to the total magnetic energy. Also, our analytics provides inspirations for scale and type manipulations of skyrmions by patterned magnetic field pluses in 2D skyrmion crystals^10^,^11^ or skyrmion-based racetrack memories^8^,^38^. Numerical simulation^38^ and experimental measurement^39^ both confirm that out-of-plane magnetic fields can control the skyrmion size. Meanwhile our results indicate the possibility that carefully designed in-plane circular magnetic field profile would induce the conversion between Néel and Bloch skyrmions.

### Field-driven planar TDW dynamics

Next we turn to planar TDW dynamics with the help of the asymptotic expansion method^19^,^20^,^21^. In this method, the dynamical behavior of a TDW is regarded as the response of its static profile to external driving factors. In this sense, it belongs to the linear response framework and suitable for traveling-wave mode of a TDW which occurs under small axial driving fields. From equations (15) and (24), the TMF strength should be finite, thus we rescale the axial driving field and the TDW propagation velocity *V* simultaneously,


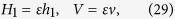


where *ε* is a dimensionless infinitesimal. We focus on the traveling-wave mode and define the traveling coordinate


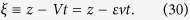


The TMF in dynamical case takes the same profile as that in static case, except for the substitution 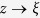 which means it moves along with the TDW center. Then we expand *θ*(*z, t*), *ϕ*(*z, t*) as follows:


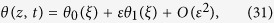



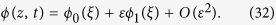


in which *θ*_0_ and *ϕ*_0_ denote the zeroth-order solution of the problem, while *θ*_1_ and *ϕ*_1_ describe the lowest order deviations with respect to the zeroth-order solutions. Put the above expansion series into scalar LLG equations (6) and (7), to the zeroth order of *ε*, we have


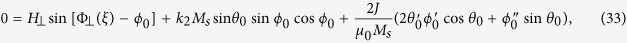


and


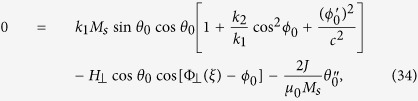


where a prime means partial derivative with respect to 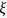 and 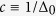. Under the comoving TMF profile (23) (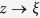), the solutions to equations (33) and (34) are just equations (20) and (28). To obtain the TDW velocity, we need to proceed to the next order.

At the first order of *ε*, we have


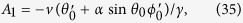


and


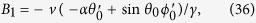


where


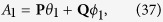



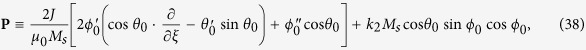



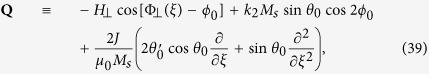


and


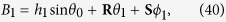



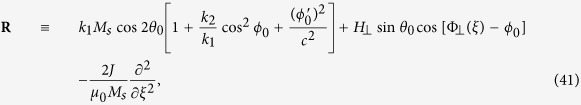



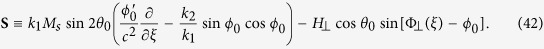


Obviously, we need to simplify operators **R** and **S** for *v*(*h*_1_) relationship. It is clear that now *θ*_0_ and *ϕ*_0_ have been fully decoupled. The partial derivative of “*B*_0_ = 0” with respect to *θ*_0_ gives


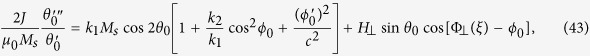


hence simplifies **R** to


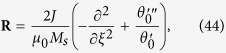


which is the same 1D self-adjoint Schrödinger operator **L** as introduced in previous works^19^,^20^,^21^. Meantime, by partially differentiating *B*_0_ = 0 with respect to *ϕ*_0_, we have


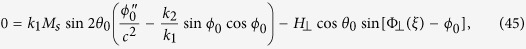


which simplifies **S** to


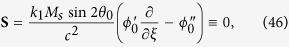


since here we are considering a complete planar TDW (*ϕ*_0_ = *const*). As a result, equation (36) becomes


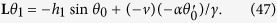


The “Fredholm alternative” requests the right hand side of equation (47) to be orthogonal to the kernel of **L** (i.e., 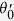) for a solution *θ*_1_ to exist. Noting that from equation (27),


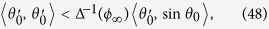


thus we finally have,


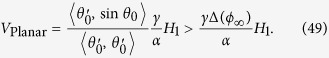


which means the planar TDW acquires a higher propagation velocity than that from the Walker analysis.

At last, it is worth of addressing the stability of the planar TDW when driven by an axial field and the feasibility of our analytical approach to TDW dynamics in rigid-body mode. In principle, the axial driving field will induce extra gyro-torque to the magnetization inside any DW. Since our planar TDW with a general tilting attitude is not the Walker-ansatz form, this extra torque would have non-uniform effect along the wire axis, hence distort the TDW tilting plane. However, in this work we focus on the field-driven dynamics in the rigid-body mode of the TDW. The critical axial field (above which the rigid-body mode collapses) is approximately the same order of magnitude as the product of the total transverse field (TMF plus transverse anisotropy field) strength and the damping coefficient. Therefore, the distortion of the TDW tilting plane in rigid-body mode should be very slight since the damping coefficient is usually pretty small. On the other hand, in this work the dynamics of a general planar TDW with the corresponding TMF pulse comoving is investigated using the asymptotic expansion method. In this approach, any slight deviation from the rigorous planar TDW is viewed as higher order expansion terms of the real solution. The TDW velocity comes from the existence condition of the first-order terms, in which only zeroth-order solutions come into real calculus, thus should not be affected by these slight distortions.

## Discussion

First, we would like to clarify that our strategy presented in this work differs from an existing one^40^. In that work, they maximized the wall velocity by optimizing the total external field with fixed strength and totally free orientation. For biaxial case, they found the optimal strategy was to let the constant-strength field always lie in the wire axis. However, they did not concentrate on the shape and attitude of the TDW (whether static or dynamic). In our work, we realize a complete planar TDW at any tilting angle by a suitable TMF pulse with fixed strength and well-designed orientation profile. The total external field also has fixed strength, but cannot freely orientate since it has a specified axial component. In brief, our strategy is not optimal for the purpose of maximizing wall velocity. However, it manipulates general planar TDWs which should have widespread applications in modern nanodevice engineering.

Second, our strategy has the challenge of statically generating and dynamically synchronizing the well-designed TMF pulse with the TDW in real experiments. For static cases, for a given TDW tilting attitude, after selecting the TMF strength and calculating the TMF orientation profile, we propose two possible methods to realize the TMF configuration in real experiments. In the first method, a series of rotatable ferromagnetic scanning tunneling microscope (STM) tips are placed along the wire axis to produce a series of localized TMF pulses. The envelope of these pulses is tuned to be the TMF profile we have calculated. In the second method, an extra nanoferromagnet with strong ferromagnetic coupling to the NW produces the effective TMF. To realize the designed TMF profile, the shape and thickness of the nanoferromagnet should be designed carefully. For dynamics, the major concern is how to effectively synchronize the TMF profile with the propagating TDW. Since the TDW velocity can be precalculated from the selected material parameters, with the help of a mature servo system, the STM tips in the first method can be tuned to rotate around the wire axis automatically to produce the comoving TMF profile.

Finally, in real experiments, the edge roughness of the wire and the resulting stochastic fields may affect the consistency between our theoretical predictions and experimental measurements. Following some pioneer works^41^,^42^, the effect of single or series of edge-notches on static profile and field-driven dynamics of planer TDWs should be an interesting issue. On the other hand, our strategy of realizing and manipulating planar TDWs can be generalized to the cases where TDW motion is driven by spin-polarized currents or temperature gradient, etc. These directions of future research would be attractive and fascinating.

## Additional Information

**How to cite this article**: Li, M. *et al*. General planar transverse domain walls realized by optimized transverse magnetic field pulses in magnetic biaxial nanowires. *Sci. Rep.* **7**, 43065; doi: 10.1038/srep43065 (2017).

**Publisher's note:** Springer Nature remains neutral with regard to jurisdictional claims in published maps and institutional affiliations.
